# miR-331-5p Affects Motility of Thyroid Cancer Cell Lines and Regulates BID Expression

**DOI:** 10.3390/biomedicines12030658

**Published:** 2024-03-15

**Authors:** Francesca Maria Orlandella, Esther Imperlini, Katia Pane, Neila Luciano, Mariantonia Braile, Anna Elisa De Stefano, Paola Lucia Chiara Iervolino, Alessandro Ruocco, Stefania Orrù, Monica Franzese, Giuliana Salvatore

**Affiliations:** 1 Dipartimento delle Scienze Mediche, Motorie e del Benessere, Università degli Studi di Napoli “Parthenope”, 80133 Naples, Italy; francescamaria.orlandella@uniparthenope.it (F.M.O.); destefanoannaelisa@gmail.com (A.E.D.S.); stefania.orru@uniparthenope.it (S.O.); 2 CEINGE Biotecnologie Avanzate Franco Salvatore S.c.a.r.l, 80145 Naples, Italy; alessandro.ruocco@virgilio.it; 3 Department for Innovation in Biological, Agrofood and Forest Systems, University of Tuscia, 01100 Viterbo, Italy; imperlini@unitus.it; 4 IRCCS SYNLAB SDN, 80143 Naples, Italy; katia.pane@synlab.it (K.P.); mariantonia.braile@synlab.it (M.B.); monica.franzese@synlab.it (M.F.); 5 Dipartimento di Scienze Biomediche Avanzate, Università degli Studi di Napoli “Federico II”, 80131 Naples, Italy; neilaluciano14@gmail.com (N.L.); paolalc89@libero.it (P.L.C.I.); 6 Dipartimento di Biologia, Università degli Studi di Napoli “Federico II”, 80126 Naples, Italy

**Keywords:** miR-331-5p, BID, thyroid carcinoma, motility

## Abstract

During tumorigenesis, miRNAs with unbalanced expression profiles can increase the threat of disease progression. Here, we focus on the role of miR-331-5p in the pathogenesis of thyroid cancer (TC). In vitro studies were conducted using TC cell lines after the forced expression and silencing of miR-331-5p. Cell proliferation and viability were analyzed via cell counts and colorimetric assays. Cell motility was analyzed via wound healing assays, Transwell migration and invasion assays, and Matrigel Matrix assays. The putative targets of miR-331-5p were unveiled via label-free proteomic screening and then verified using Western blot and luciferase assays. Expression studies were conducted by interrogating The Cancer Genome Atlas (TCGA). We found that ectopic miR-331-5p expression reduces TC cell motility, while miR-331-5p silencing induces the opposite phenotype. Proteomic screening revealed eight putative downregulated targets of miR-331-5p, among which BID was confirmed as a direct target. TCGA data showed the downregulation of miR-331-5p and the upregulation of *BID* in TC tissues. In summary, deregulation of the miR-331-5p/BID axis could enhance the aggressiveness of TC cell lines, providing new insights into the mechanisms of the progression of this disease and suggesting a potential role of the component factors as possible biomarkers in TC tissues.

## 1. Introduction

Thyroid cancer (TC) is the most frequently diagnosed endocrine-related malignancy, accounting for about 3–4% of all human cancers [1]. Based on its histology, TC originating from follicular cells can be classified as either well-differentiated carcinoma (WDTC), which includes papillary (PTC), follicular (FTC), and Hürthle cell (HCTC), or undifferentiated carcinoma [2]. WDTC is more likely to occur in females. It usually has a good prognosis, and treatment is based on surgery, thyroid ablation, and radioactive iodine therapy [3,4,5,6,7,8]. Undifferentiated or anaplastic thyroid carcinoma (ATC) represents one of the rarest and most aggressive human cancers. It can develop from pre-existing WDTC following additional mutational events. Novel therapeutic strategies would be positive news for the treatment of the aggressive forms of TC, which are unresponsive to radioactive iodine cures and progress rapidly to other organs [9,10,11].

The genomic characterization of TC has not only highlighted that BRAF and RAS mutations are the key drivers of PTC [12,13] but also that additional genetic alterations are necessary for evolution to the anaplastic phenotype, such as mutations in TP53, the TERT promoter, cell cycle genes, histone methyltransferases as well as components of the PI3K pathway and mismatch repair (MMR) signaling [14,15,16].

Considering its complexity, unbalanced microRNA (miRNA) expression is closely associated with the biological process of TC progression [17,18,19] and drug resistance [20,21]. miRNAs are endogenous single-stranded non-coding molecules capable of influencing several signaling pathways via their ability to regulate hundreds of target genes acting as tumor suppressors or oncogenes in a tissue-dependent manner [22]. Furthermore, since circulating miRNAs are stable molecules in plasma, they could represent potential candidates to be used as cancer biomarkers and prognostic indicators for TC [23,24].

In this study, we sought to investigate miR-331-5p, a member of the miR-331 family [25], because it plays a potential tumor suppressor role in several human cancers, i.e., leukemia and lung, prostate, gastric, breast, hepatocellular, and colorectal cancers. However, its role in the pathogenesis of thyroid malignancy remains unknown. Furthermore, miR-331-5p is one of the miRNAs that we found to be downregulated in a PTC (TPC-1) cell line ectopically expressing the transcription factor TWIST1, a master regulator of the epithelial-to-mesenchymal transition [21].

In leukemia, the downregulation of miR-331-5p has been associated with chemotherapy resistance [26]. Recently, the deregulation of miR-331-5p was reported in lung cancer, where it appears to be one of the key mediators of the anti-invasive activity of curcumin by regulating the expression of MAPK pathway components and TGFB2 [27]. In prostate cancer, miR-331-5p regulates the expression of UGT2B15, a gene correlated with a higher risk of prostate cancer insurgence [28]. Further, the overexpression of miR-331-5p in gastric cancer cell lines impairs cell proliferation, motility, and glycolysis by directly targeting PFKFB3 [29].

Concerning the potential application of miR-331-5p as a biomarker, higher miR-331-5p expression was observed in the plasma of women with breast cancer compared to a control group, where it correlated with a lower DNA repair capacity [30]. Based on an in silico analysis of hepatocellular carcinomas deposited in TCGA, it was determined that miR-331-5p is negatively correlated with overall survival and a predictor of progression-free survival [31]. In addition, the potential use of miR-331-5p as a biomarker in hepatocellular carcinoma was more recently supported in a study by Yang and colleagues in which high expression of this miRNA was correlated with a higher survival rate in these patients [32]. Finally, miR-331-5p circulating plasma levels could be useful for discriminating patients with colorectal adenomas from those with polyps and control subjects, suggesting the potential of this miRNA as a tool in screening [33].

Thus, overall, there are a limited number of studies on miR-331-5p, and its role in cancer remains elusive. For this reason, the primary goal of the present study was to assess the effects of miR-331-5p silencing and its forced expression in human TC cell lines toward providing new evidence of its role in TC pathogenesis. In silico studies were also carried out to explore the expression levels of miR-331-5p in normal and TC tissues.

Our findings reveal that miR-331-5p is downregulated in TC tissues compared with normal thyroid tissues from the TCGA dataset; functional experiments suggested that miR-331-5p deregulation affects TC cell migration and their invasion ability. Deeper analyses revealed that BID is a direct target of miR-331-5p.

Overall, the obtained data highlight the miR-331-5p/BID axis as a potential theragnostic marker for TC.

## 2. Materials and Methods

### 2.1. RNA Extraction and qRT-PCR

Total RNA was isolated from cell lines using TRIzol Reagent (Thermo Fisher Scientific, Waltham, MA, USA) and quantified using a NanoDrop spectrophotometer (Thermo Fisher Scientific).

For miRNA detection, 10 ng of total RNA was reverted using a TaqMan MicroRNA RT Kit (Thermo Fisher Scientific) according to the manufacturer’s instructions. Next, the expression levels of the mature form of miR-331-5p and the endogenous control (U6 small nuclear RNA) were determined via qRT-PCR using a specific probe, primers, and TaqMan Universal Master Mix II (Thermo Fisher Scientific).

qRT-PCR was performed in triplicate for each sample, and the relative gene expression was reported as a fold change and was calculated using the 2^−ΔΔCt^ method.

### 2.2. Cell Cultures

The human papillary (TPC-1) and anaplastic (8505C and CAL62) thyroid cancer cell lines were cultured in Dulbecco’s modified Eagle’s medium (DMEM) supplemented with 10% fetal bovine serum (FBS), L-glutamine, and penicillin/streptomycin and grown at 37 °C with 5% CO_2_. All the reagents were purchased from Thermo Fisher Scientific.

The ATC cell lines (8505C and CAL62) were obtained from DSMZ (Deutsche Sammlung von Mikroorganismen und Zellkulturen GmbH, Braunschweig, Germany). The TPC-1 cell line was from M. Nagao (Carcinogenesis Division, National Cancer Center Research Institute, Tokyo, Japan).

### 2.3. Cell Transfections

Plasmids expressing the precursor of miR-331-5p (HmiR0192-MR04) and the precursor miRNA scrambled control (CMIR0001-MR04, miR-Null) as well as plasmids inhibiting miR-331-5p (HmiR-AN0419-AM01) and the miRNA inhibitor scrambled control (CMIR-AN0001-AM01, Anti-miR-Null) were purchased from Gene-Copoeia (Nivelles, Belgium).

For stable transfection, Lipofectamine 2000 transfection reagent (Thermo Fischer Scientific, Waltham, MA, USA, Catalog number: 11668500) was used. Following puromycin (Sigma-Aldrich, St. Louis, MO, USA) selection, one mass population was selected based on miR-331-5p expression.

Lipofectamine 2000 transfection reagent was adopted for transient transfection of the abovementioned plasmids according to the manufacturer’s instructions. At the end of the experiments, the transfection efficiency was evaluated using qRT-PCR.

### 2.4. Cell Proliferation Assay

For cell counting, stably transfected cells (TPC-1, CAL62, and 8505C) were seeded at a density of 3 × 10^4^ in 6-well plates and incubated at 37 °C. After 24, 48, 72, and 96 h, cells were collected via trypsinization and counted using a TC10 automated cell counter (Bio-Rad, Hercules, CA, USA). Transiently transfected cells (TPC-1, CAL62, and 8505C) were collected via trypsinization and counted using a TC10 automated cell counter (Bio-Rad) at the end of the experiments.

The CellTiter 96 AQueous One Solution Cell Proliferation Assay (MTS, Promega, WI, USA), a colorimetric method, was used to determine the number of viable cells. Briefly, 1 × 10^3^ cells were seeded in a 96-well plate, and after 24, 48, 72, and 96 h, 20 µL of MTS solution was added to each well and incubated at 37 °C for 30 min. Then, the absorbance at 490 nm was recorded using an automatic microplate reader (Microplate Manager Version 5.2.1, Bio-Rad).

### 2.5. Cell Migration Assay

The cell migration ability was determined using wound healing and Transwell assays as previously described [34].

For the wound healing assay, transfected TC cells were seeded in 6-well culture plates and incubated for 24 h at 37 °C to reach confluency. Following this incubation, a scrape was made in the confluent cellular monolayer. The scrape area was monitored at 5× magnification using phase-contrast microscopy (Leica DM4000 microscope) and was photographed from immediately after wound incision until closure. The wound area was measured using ImageJ software version 1.53t and expressed as a percentage of wound closure compared to control cells.

For the Transwell migration assay, 1 × 10^5^ cells were resuspended in 100 µL of DMEM with 5% FBS and seeded in an upper chamber containing a polycarbonate membrane filter with an 8 μm pore size (Costar, Cambridge, MA, USA). In the lower well, 500 μL of DMEM with 10% FBS was added and used as a chemoattractant [35]. After 24 h for the TPC-1 cell line or 48 h for the CAL62 cell line, non-migratory cells were removed using a cotton swap, while migrated cells were fixed with 11% glutaraldehyde solution (Merck KGaA, Darmstadt, Germany), colored with crystal violet, photographed, and quantified at O.D. 550 nm (Microplate Manager Version 5.2.1, Bio-Rad Laboratories, Hercules, CA, USA).

### 2.6. Cell Invasion Assay

To study the invasion ability of the cells, Matrigel Matrix and 3D Matrigel assays were performed.

For the Matrigel Matrix assay, 1 × 10^5^ cells in DMEM with 5% FBS were plated in the upper well of a pre-hydrated polycarbonate membrane filter with an 8 μm pore size coated with 35 μg of reconstituted extracellular Matrix (Matrigel, BD Biosciences, San Jose, CA, USA). Afterwards, cells were incubated for 24 h (TPC-1 and 8505C) or 48 h (CAL62). Subsequently, 11% glutaraldehyde solution (Sigma-Aldrich) was used to fix the cells that invaded the polycarbonate membrane, and such cells were stained with crystal violet, eluted, and quantified using a spectrophotometer (O.D. 550 nm).

In TPC-1 cells, invasion was also investigated using 3D Matrigel assays according to the manufacturer’s instructions. Briefly, 45 µL of Matrigel Basement Membrane Matrix (BD Biosciences, San Jose, CA, USA) was added to Nunc Lab-Teck chambered coverglasses (Thermo Fischer Scientific) and incubated for 30 min at 37 °C. Then, 5 × 10^5^ thyroid cancer cells were resuspended in 500 µL of DMEM with 10% FBS enriched with 2% Matrigel and plated on all chambered coverglasses. Cells were incubated for two weeks and photographed at different times (4, 8, and 12 days).

### 2.7. Transendothelial Migration Assay

In order to assess the ability of CAL62 cells to invade the endothelium, normal human umbilical vein endothelial cells (HUVEC, 1 × 10^5^) grown in Endothelial cells Growth Media (EGM-2) were added to a Transwell insert coated with fibronectin and then treated with 20 ng/mL TNF-α for 18 h (Cell Biolabs, San Diego, CA, USA). Next, 1 × 10^5^ CAL62 cells transfected with miR-331-5p or a control plasmid were seeded on an endothelial cell monolayer for 24 h. Following this incubation, invasive tumor cells that had migrated in the outer chamber through the HUVEC monolayer were stained and quantified using a spectrophotometer at O.D. 550 nm.

### 2.8. Western Blot

Proteins were lysed in JS Buffer and quantified with a spectrophotometer using the Bradford method (Bio-Rad). Protein lysates were separated via SDS-PAGE in a 14% polyacrylamide gel and then transferred to nitrocellulose membranes. After blocking, the membranes were probed overnight at 4 °C with the following antibodies: anti-BID (B-3) monoclonal antibody (sc-373939, Santa Cruz Biotechnology, Dallas, TX, USA) and anti-α-TUBULIN (T9026) monoclonal antibody (Merck KGaA). After washing, species-specific secondary antibodies (Bio-Rad) coupled to horseradish peroxidase were added to the blot. Antigen–antibody complexes were visualized using a chemiluminescence detection solution (Thermo Fisher Scientific) and analyzed on a Bio-Rad Chemidoc (Bio-Rad).

### 2.9. Label-Free Proteomic Screening of Transfected CAL62 Cells

CAL62/miR-331-5p and CAL62/miR-Null cells were separately lysed in a modified RIPA buffer (150 mM NaCl, 50 mM Tris-HCl (pH 7.5), 1 mM EDTA, 1% Triton X-100, and protease inhibitors). For each sample, cell debris was removed via centrifugation, and the protein concentration was then measured using a Bradford assay [36]. Protein extracts were separated on 10% polyacrylamide via SDS-PAGE. After staining with Colloid Blue Stain Reagent (Thermo Fisher Scientific, Waltham, MA, USA), gel images were acquired using a GS-800 Calibrated Densitometer scanner (Bio-Rad, Hercules, CA, USA). Then, the gel lanes were manually cut into slices and further processed as previously described [37]. Enzymatic digestion was carried out with 10 ng/mL modified trypsin (Promega, Madison, MI, USA). Peptide mixtures were extracted and resuspended in 0.2% (*v*/*v*) formic acid for mass spectrometry (MS) analysis.

MS analysis was conducted using an LTQ-Orbitrap XL (Thermo Fisher Scientific, Bremen, Germany) equipped with a nanoEASY II Nanoseparations chromatographic system (75 μm–L 20 cm, column, Thermo Scientific, Bremen, Germany). MS peptide analysis was performed as previously described [38]. Raw MS/MS data were converted into Mascot generic format (.mgf) and processed using Proteome Discoverer platform version 1.4 (Thermo Fisher Scientific, Waltham, MA, USA) interfaced with an in-house Mascot server version 2.3 (Matrix Science, London, UK). The search parameters for protein identification were UniProt as the database (Homo sapiens taxonomy); trypsin as a specific proteolytic enzyme; one missed cleavage allowed; 10 ppm precursor tolerance and 0.6 Da fragment ion tolerance; carbamidomethylation of cysteine as a fixed modification; and N-terminal glutamine conversion to pyro-glutamic acid and oxidation of methionine as variable modifications. Identification was allowed with a minimum of two peptides, each characterized by an individual Mascot score > 19. Each protein quantification was estimated according to the label-free approach based on normalized spectral counts (SpC). The obtained protein datasets were imputed in order to replace any missing values, with the detection limit defined as 1/5 of the minimum positive value of each variable, as previously reported [39]. Finally, the relative protein abundance was expressed as a fold change (FC), calculated as the log2 of the SpC ratio between the sample (CAL62/miR-331-5p cells) and the control (CAL62/miR-Null cells). In the present study, proteins with FC values ≥ 1.20 or ≤−1.20 were considered differentially represented in the CAL62/miR-331-5p and CAL62/miR-Null cell lines. The 1.20 cut-off was previously established in several differential proteomics studies, regardless of the specific proteomics technique applied [40,41,42]. The mass spectrometry proteomics data were submitted to the ProteomeXchange Consortium [43] via the PRIDE [44] partner repository with the dataset identifier PXD039884.

### 2.10. In Silico Analysis

Differential gene expression analysis was performed using IPA accessed on 18 December 2023 v. 107193442 (QIAGEN Inc., https://www.qiagenbioinformatics.com/products/ingenuitypathway-analysis) using RNA-seq data for the TCGA thyroid cohort generated by the TCGA Research Network (https://portal.gdc.cancer.gov/projects/TCGA-THCA) and processed by R software (version 4.1.3) [45]. Data were extracted with gene-level quantification based on Human Genome build 38 with GenCode Release 33. BRAF-RAS classes were defined according to [12].

To predict the potential miRNA–mRNA binding site, the miRWalk (http://mirwalk.umm.uni-heidelberg.de/) [46] was accessed on 27 December 2023; the RNAhybrid version 2.2 (https://bibiserv.cebitec.uni-bielefeld.de/rnahybrid) [47] and the STarMir (https://sfold.wadsworth.org/cgi-bin/starmirWeb.pl) [48] online tools were accessed on 11 January 2024.

### 2.11. Luciferase Assay

To verify if BID is a direct target of miR-331-5p in TC cells, we performed a luciferase assay. To this aim, we used a commercial plasmid (LightSwitch™, Product #S809507) in which the luciferase-coding sequence is fused to the 3′ UTR of BID under the control of a constitutive promoter. The empty vector (EMPTY_3UTR, Product #S890005), containing only the luciferase gene was used as a positive control. The 3′ UTR of beta ACTIN (ACTB_3UTR Product # S804753), a housekeeping gene cloned downstream of the luciferase gene, was co-transfected in cells and used to normalize the transfection efficiency.

Briefly, 1 × 10^3^ cells (CAL62/miR-331-5p and CAL62/miR-Null) were plated in triplicate for each condition in a 96-well plate, and the next day, the cells were transfected with 50 ng/well of the commercial DNA plasmids described above. FuGENE HD was used as a transfection reagent at a ratio of 3:1 with the DNA plasmid according to the manufacturer’s instructions. Then, 24 h after the transfections, the luciferase signal was measured by incubating each well with 100 μL of LightSwitch Assay Solution (Catalog number: 32031) for 30 min at room temperature, as indicated in the manufacturer’s protocol. Subsequently, the plate was read for 2 s using a luminometer (EnSpire^®^ Multimode Plate Reader, PerkinElmer, Waltham, MA, USA).

All reagents were purchased from SwitchGear Genomics (La Hulpe, Belgium).

### 2.12. Statistical Analysis

Statistical analyses for experimental validation were elaborated using GraphPad Prism 9 software (La Jolla, CA, USA). The in vitro results were represented as the means and the standard errors of the means (±SEMs), and the statistical analysis was calculated using Student’s *t*-test.

The Wilcoxon test was used for comparisons between two groups, and the Kruskal–Wallis test was used to compare more than two groups. *p* values were considered statistically significant when *p* < 0.05.

Spearman’s correlation analysis between the gene and miRNA expression levels was performed using TCGA-THCA RNA-seq and miRNA-Seq data, respectively, for primary tumor samples.

## 3. Results

### 3.1. miR-331-5p Influences the Motility Ability of Thyroid Cancer Cell Lines

To determine the biological phenotype induced by miR-331-5p in human TC cell lines, we modulated its expression in a panel of ATC (CAL62 and 8505C) and PTC (TPC-1) cell lines using both stable and transient transfections.

Thus, we started studying the ectopic expression of miR-331-5p by stably transfecting a plasmid carrying an miR-331-5p precursor and an miRNA scrambled control plasmid (miR-Null) into TPC-1 and CAL62 cells. In parallel, we increased the miR-331-5p expression in the 8505C cell line via transient transfection. After antibiotic selection for stable transfection (Figure S1A,B) and 48 h after transient transfection (Figure S1C), qRT-PCR was performed to verify the ectopic expression of miR-331-5p.

**Figure 1:**
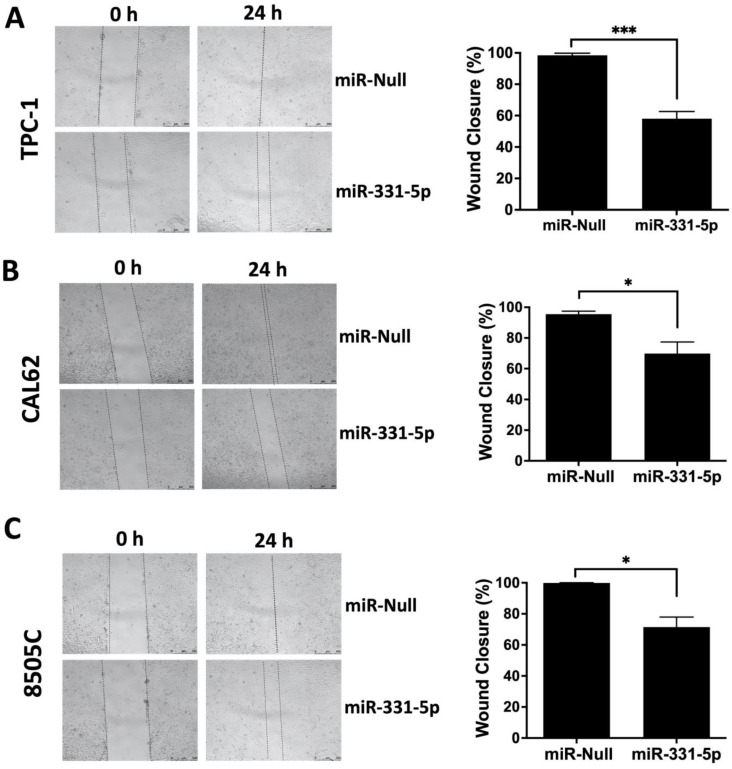
The ectopic expression of miR-331-5p impairs the migration rate of thyroid cancer cell lines. The migration ability was investigated via wound healing assays of TPC-1 (**A**) and CAL62 (**B**) cells stably transfected with a plasmid expressing the precursor of miR-331-5p or the control plasmid (miR-Null). The migration ability was further monitored via wound healing in an ATC cell line (8505C) transiently transfected with these plasmids for 48 h (**C**). The area of the gap was measured from the initial time (0 h) until the closure. The percentage of the wound closure was calculated in relation to the area value of the initial scratch. The lines define the area of the gap. Scale bar: 500 μm. All the experiments were performed at least three times, and the data are presented as mean ± SEM. * *p* < 0.05; *** *p* < 0.001.

Then, we examined the migration ability of these cells by performing a wound closure assay. As shown in Figure 1, the forced expression of miR-331-5p significantly reduced the capability of the TPC-1 (A) and CAL62 (B) cells to migrate into the wound compared to cells transfected with the control vector (miR-Null). A similar result was also obtained when we transiently overexpressed miR-331-5p in 8505C cells (Figure 1C).

**Figure 2:**
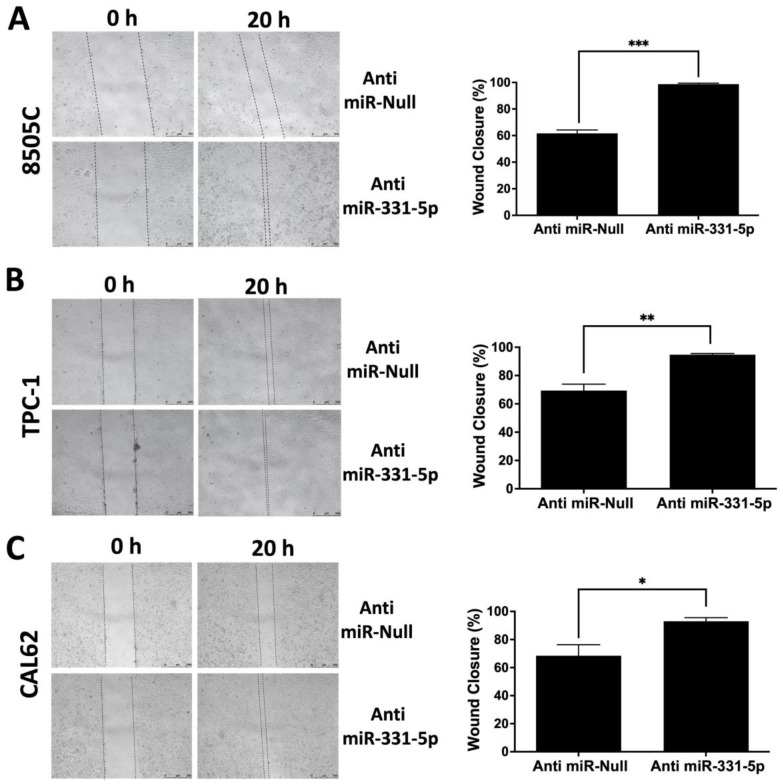
Silencing of miR-331-5p increased the migration rate of thyroid cancer cell lines. A scratch was inflicted on a confluent monolayer of 8505C cells with stably silenced miR-331-5p expression or with the control plasmid (Anti-miR-Null) (**A**). TPC-1 (**B**) and CAL62 (**C**) cell lines were transiently transfected with the indicated plasmids, and the next day a scratch was inflicted with a sterile pipette tip in each well. Photos were taken immediately after the wound incision (0 h) and at the moment of wound closure (20 h). The lines define the area of the gap. Scale bar: 500 μm. Each column represents the mean of at least three experiments ± SEM. * *p* < 0.05; ** *p* < 0.01; *** *p* < 0.001.

Next, to confirm our findings, we performed a mirrored experiment in which we silenced endogenous miR-331-5p by stably transfecting its inhibitor (Anti-miR-331-5p) and the relative control plasmid (Anti-miR-Null) into 8505C cells. As reported in Figure 2A, the silencing of miR-331-5p in the 8505C cell line increased its ability to migrate into the wound compared to the 8505C/Anti-miR-Null cells. Transient silencing of miR-331-5p in TPC-1 (Figure 2B) and CAL62 (Figure 2C) cells also led to increases in motility.

The efficiencies of the miR-331-5p silencing by the stable (A) and transient (B,C) transfections are reported in Figure S2. The modulation of miR-331-5p in TC cells did not significantly influence the proliferation rate at the times or in the experimental conditions that were tested (Figures S3 and S4).

**Figure 3:**
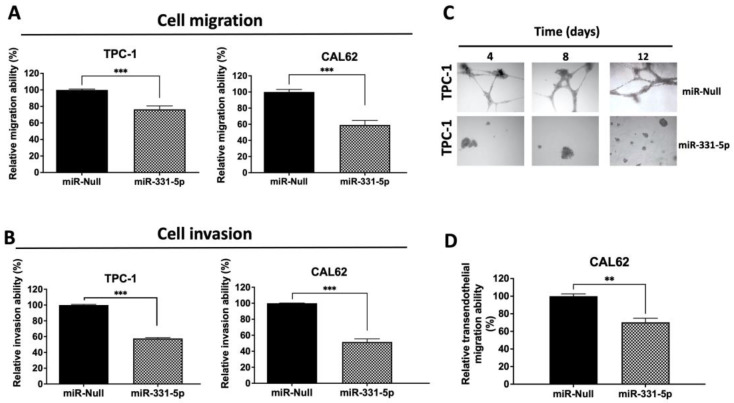
Modulation of miR-331-5p expression affects the invasive phenotype of thyroid cancer cell lines. Cell migration (**A**) and cell invasion (**B**) were evaluated by plating stably transfected TPC-1 and CAL62 cells onto the Transwell insert (8.0 µm pore diameter) in the absence (**A**) or presence (**B**) of Matrigel Matrix and allowing them to migrate/invade for 24 h (TPC-1) or 48 h (CAL62). After incubation, cells were stained with crystal violet and quantified through the elution of the colorant and spectrophotometric detection (O.D. 550 nm). The number of migrated cells was reported as fold change compared to control cells (set equal to 100%). (**C**) Assessment of TPC-1 cell morphology after forced expression of miR-331-5p in a 3D cell culture model. After plating, stably transfected TPC-1 cells were monitored and photographed at different times (4, 8, and 12 days). (**D**) CAL62/miR-331-5p and CAL62/miR-Null cells were plated on the upper side of an insert coated with fibronectin and a monolayer of HUVEC cells. After 24 h, the invasive cells were stained with crystal violet and quantified spectrophotometrically at O.D. 550 nm (shown as a percentage). Data are represented as mean ± SEM. ** *p* < 0.01; *** *p* < 0.001.

We further characterized the motility phenotypes of the PTC (TPC-1) and ATC (CAL62 and 8505C) cell lines overexpressing miR-331-5p by evaluating cell migration into a Transwell insert and by evaluating cell invasion into a Transwell insert coated with Matrigel. As shown in Figure 3, TPC-1 and CAL62 cells stably transfected with miR-331-5p exhibit significant reductions in their migration (A) and invasion (B) abilities in the chambers in the absence or presence of Matrigel Matrix, respectively, compared to the control transfected cells.

In the mirrored experiment, as expected, the silencing of miR-331-5p in 8505C cells increased the invasive capability of these cells compared to the 8505C/Anti-miR-Null cells (Figure S5).

As further proof, the invasive capacity of the transfected cells was also assessed using a three-dimensional (3D) invasion assay. To this end, TPC-1 cells were seeded within complex 3D microenvironments and monitored after 4, 8, and 12 days. Figure 3C shows that TPC-1/miR-331-5p cells were smaller and displayed a lower invasive ability in 3D Matrigel compared to cells transfected with the relative control at all time points that were analyzed.

Taken together, these data indicate that the modulation of miR-331-5p expression influences TC cell invasion.

### 3.2. Overexpression of miR-331-5p Reduces Transendothelial Migration of CAL62 Cells

Finally, since the acquisition of transendothelial migration ability is an important step in cancer progression and metastasis, we investigated the role of miR-331-5p in this biological process.

Hence, CAL62/miR-331-5p and CAL62/miR-Null cells were seeded on a thin HUVEC monolayer for 24 h. Then, the invasive tumor cells were stained and spectrophotometrically quantified. We observed that forced miR-331-5p expression significantly inhibits the ability of CAL62 cells to invade the endothelial cells in comparison to control cells (Figure 3D).

### 3.3. Unveiling miR-331-5p Targets

Next, we focused on identifying the targets of miR-331-5p in thyroid cancer cells. Thus, we carried out label-free proteomic analysis to unveil the protein expression profiles of CAL62/miR-331-5p and CAL62/miR-Null cells. This analysis revealed 39 proteins that were differentially expressed (31 upregulated and 8 downregulated) among the two groups with fold changes ≥ 1.20 or ≤ −1.20 (Table S1).

Among the predicted targets of miR-331-5p, we focused on the eight proteins (ATP11A, BID, CHMP2B, COMMD10, NLN, NUP54, USP24, and ZC3H18) downregulated in CAL62/miR-331-5p cells versus control cells (Table S1).

**Figure 4:**
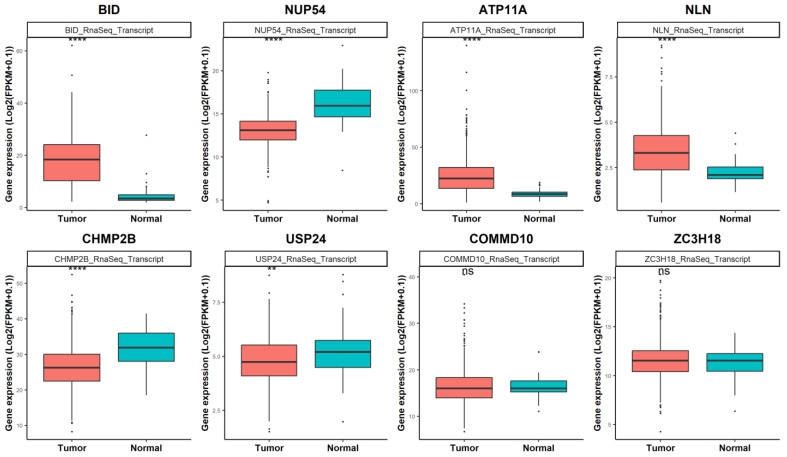
Differential gene expression analysis of the putative targets of miR-331-5p in TCGA thyroid cohort (TCGA-THCA). The eight genes downregulated in CAL62/miR-331-5p cells were investigated in thyroid cancer tissues (N = 510) compared to normal thyroid tissues (N = 58) deposited in TCGA. Statistical significance *p* values ** *p* < 0.01; **** *p* << 0.001 and ns according to a Wilcoxon rank sum test with continuity correction.

To ascertain the expression levels of these genes in a large cohort of human thyroid cancer and normal thyroid tissues, we performed in silico analysis using gene expression data (RNA-seq) from The Cancer Genome Atlas Thyroid Cancer project (TCGA-THCA). Figure 4 shows that for six of eight putative downregulated miR-331-5p targets, the gene expression changed in a statistically significant manner in 510 tumors (red box) compared to 58 normal thyroid tissues (turquoise box). Among the eight genes, *BID* had the highest fold change (3.97, *p* = 4.45 × 10^−27^) in tumors compared to normal tissues.

**Figure 5:**
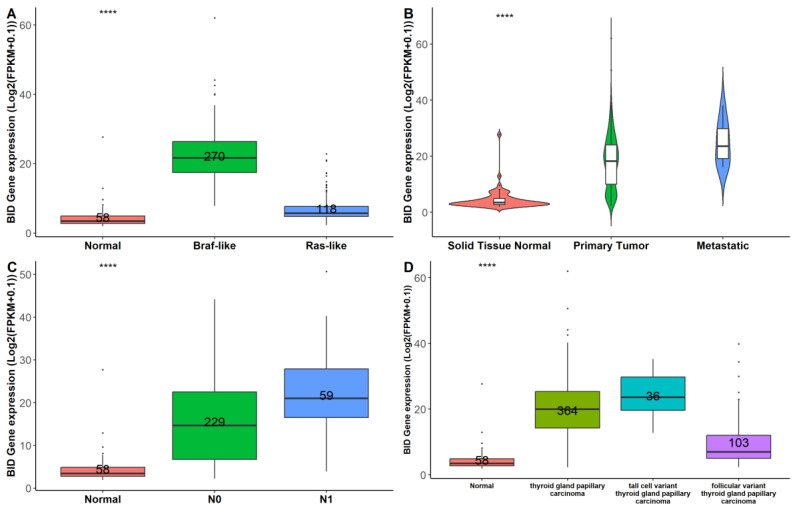
Analysis of BID expression across the TCGA-THCA cohort sorted by genetic and clinicopathologic characteristics. BID expression changes, comparing normal tissues and tissues from thyroid tumor patients harboring Braf-like and Ras-like mutations, according to BRAF and RAS mutation status reported in [12] (**A**); violin plot of normal (N = 58), tumor (N = 502), and metastatic (N = 8) thyroid tissues (**B**); normal tissues versus tumors stratified by nodal metastasis status (**C**); and normal tissues versus histological tumor subtypes (**D**). Numbers within each box represent the sample sizes. **** *p* << 0.001.

To gain further insight into the role of BID in thyroid carcinogenesis, we assessed its expression level across the TCGA-THCA cohort based on genetic and clinicopathologic characteristics. Interestingly, there was significant overexpression of *BID* in Braf-like thyroid tumors (n = 270) compared to normal (n = 58) and Ras-like (n = 118) thyroid tissues (Figure 5A). In addition, the highest expression of *BID* was observed in metastatic thyroid tumors (n = 8) based on comparison to 502 thyroid tumor tissues and 58 normal thyroid tissues (*p* = 1.11 × 10^−26^) (Figure 5B).

In agreement with the fact that the overexpression of *BID* could be correlated with a more aggressive phenotype, we observed that *BID* expression was lower in the absence of nodal metastasis (N0) compared to N1 disease (Figure 5C) as well as in the follicular histological subtype compared to the classical and tall thyroid papillary carcinoma subtypes (Figure 5D), which are generally associated with worse clinical outcomes [49].

### 3.4. miR-331-5p Expression Is Downregulated in TC Tissues and Inversely Correlated with BID

**Figure 6:**
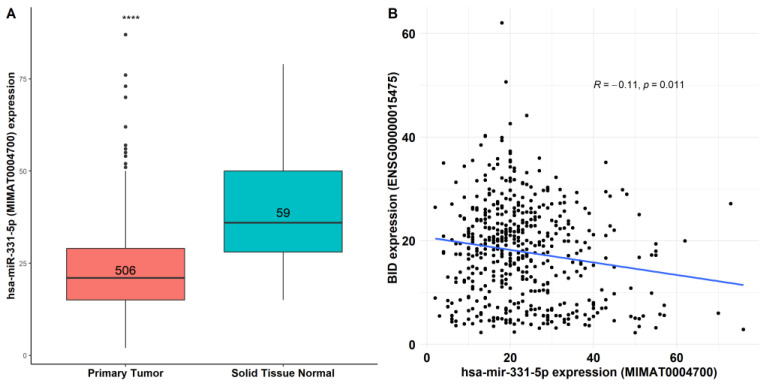
Co-expression analysis of miR-331-5p and BID in thyroid cancer tissues. (**A**) Differential expression of human miR-331-5p (MIMAT0004700) between 506 thyroid cancer tissues and 59 normal thyroid tissues deposited in TCGA-THCA (*p* = 1.1 × 10^−14^). (**B**) Co-expression analysis of miR-331-5p versus BID mRNA (ENSG00000015475), **** *p* << 0.001.

To determine the expression of miR-331-5p in TC tissues, we used the TCGA-THCA cohort, revealing that miR-331-5p is significantly downregulated (fold change of about 0.6, *p* value = 1.1 × 10^−14^) in TC compared to normal thyroid tissues (Figure 6A).

Furthermore, we investigated the possible correlations of hsa-miR-331-5p (MIMAT0004700) and the *BID* mRNA target (ENSG00000015475) across the thyroid cancer tumors deposited in TCGA-THCA, finding an inverse association (*r* = −0.11, *p* = 1.1 × 10^−2^) between miR-331-5p and BID (Figure 6B).

### 3.5. miR-331-5p Targets BID in Thyroid Cancer Cell Lines

**Figure 7:**
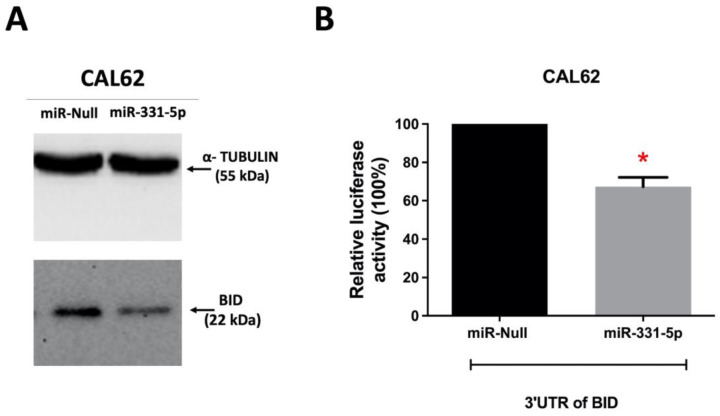
BID is a direct target of miR-331-5p in thyroid cancer cells. (**A**) Protein levels of BID in CAL62/miR-Null and CAL62/miR-331-5p cells were analyzed via immunoblotting using α-TUBULIN as an endogenous control. (**B**) Luciferase vector containing the wild type of the 3′ UTR of BID was transfected into CAL62/miR-Null and CAL62/miR-331-5p cells. The 3′UTR of beta ACTIN, cloned downstream of the luciferase reporter, was co-transfected and used to normalize the luminescence values of the 3′UTR of BID across the cells. After incubation, the luciferase activity was measured and reported as relative luciferase activity expressed as a percentage. Data are represented as mean ± SEM of two independent experiments performed in triplicate. * *p* < 0.05.

Subsequently, we investigated the expression level of BID protein in the same cells in which we conducted proteomic analysis. We found that in CAL62/miR-331-5p cells, BID expression was downregulated compared to CAL62/miR-Null cells (Figure 7A), suggesting that the interaction between miR-331-5p and BID results in translational repression. Further, since the potential interaction between miR-331-5p and the 3′ UTR of BID was predicted by the online tools miRWalk, RNAhybrid, and STarMir (Figure S6), we performed a luciferase activity assay to verify the direct physical interaction between the targeting sequence of miR-331-5p and the 3′ UTR of BID mRNA. In this set of experiments, a plasmid generated with the 3′ UTR of BID and the luciferase reporter gene was transfected into CAL62/miR-Null and CAL62/miR-331-5p cells and incubated for 24 h. After this, the luciferase activity was measured. Figure 7B shows that there is considerably lower luciferase activity in CAL62/miR-331-5p cells compared to CAL62/miR-Null cells. This result demonstrates that BID is a direct target of miR-331-5p.

## 4. Discussion

The mortality of thyroid cancer generally occurs in clinically aggressive PTC that becomes radioiodine refractory and in ATC patients that show loco-regional or distant metastases at the time of diagnosis [7,50]. ATC is also one of the most aggressive human cancers because ATC cells lack the typical features characteristic of thyroid gland differentiation and thereby become resistant to all therapies [10,11,14,51].

Understanding the molecular events that occur in the TC transformation will facilitate the development of novel diagnostic and therapeutic approaches, leading to personalized medicine and customized treatment, which represents the main goal in the field of oncology research.

In this context, there is increasing evidence suggesting that microRNAs (miRNAs) are involved in neoplastic transformation and are thus responsible for the tumor aggressiveness, secondary recurrence, metastasis, and drug resistance of different human cancers, including thyroid neoplasia [20,52,53,54]. Indeed, many studies have reported that miRNAs, by binding to complementary sequences in the 3′ untranslated regions (UTRs) of target messenger RNAs (mRNAs), influence cell proliferation, apoptosis, the cell cycle, immune responses, and cell motility in healthy human cells and cancer cells.

Among the deregulated miRNAs reported in the literature, the miR-331 family is implicated in the neoplastic transformation and the development of metastases. The human miR-331 gene is located on chromosome 12q22 and gives rise to two mature miRNAs: miR-331-3p and miR-331-5p.

Concerning the biological functions of miR-331-3p in the tumorigenesis process, it has been reported that this miRNA exerts both tumor suppressor and tumor promoter roles. In particular, its oncosuppressive function has been recognized in gastric [55], non-small-cell lung [56], breast [57], ovarian [58], urothelial [59], and cervical [60,61] cancers. Additionally, in thyroid cancer, it has been reported that the overexpression of miR-331-3p results in the inhibition of cell proliferation and motility using PTC cells [62].

To our knowledge, the role of miR-331-5p in human cancer, particularly in thyroid carcinogenesis, is poorly investigated. We previously found that miR-331-5p is one of the miRNAs downregulated in TPC-1 overexpressing TWIST1 cells compared to TPC-1 cells transfected with control plasmid [21].

Here, we discovered that miR-331-5p is downregulated in thyroid cancer tissues, and we demonstrated that miR-331-5p affects the motility abilities of PTC (TPC-1) and ATC (CAL62 and 8505C) cell lines. Notably, miR-331-5p modulation did not significantly affect cell proliferation in our model system under the tested conditions. This result may be due to the fact that different proteins and pathways are involved in proliferation and migration processes [63]. It is also possible that an effect of miR-331-5p on proliferation could be detected at longer time points.

Finally, through label-free proteomic analysis, we investigated the molecular targets governed by miR-331-5p, and we demonstrated through a luciferase assay that BID is a direct target. BID is significantly overexpressed in thyroid cancer patients, where it is inversely correlated with miR-331-5p.

*BID* is located on chromosome 22, and it encodes a pro-apoptotic protein belonging to the BCL-2 family [64,65]. Following DNA damage, BID interacts with BAX protein, activating the intrinsic apoptotic pathway and exerting a crucial role in the intra-S-phase checkpoint [66]. Moreover, it was reported that the deletion of BID in hepatocytes induces the inhibition of cell death and protects animals from tumorigenesis [67]. In accordance with our results, Gunda and colleagues, by analyzing the human PTC expression profiling of apoptotic genes, found that BID was upregulated in PTC samples versus matched normal thyroid tissues [68]. These data are in accordance with ours obtained from TCGA-THCA, in which BID was found to be overexpressed in TC tissues compared to normal thyroid tissues. Moreover, Gunda and colleagues also suggested that BID is one of the genes responsible for the apoptotic effects of lexatumumab, a monoclonal antibody that specifically activates TRAIL-receptor 2, inducing cell death in thyroid cancer cells [68]. The importance of the lexatumumab/BID interaction in human cancers was also reported in other papers, where lexatumumab induced apoptosis through the activation of BID in non-Hodgkin’s lymphoma [69], melanoma [70], and liver tumor cells [71].

Notably, in agreement with our data, BID was recently identified as a prognostic factor in PTC [72].

## 5. Conclusions

In this study, we investigated the role of miR-331-5p in TC. We found that miR-331-5p overexpression reduces TC cell migration and invasion, while its inhibition induces the opposite effect. Label-free proteomic analysis together with Western blot and luciferase assays revealed that miR-331-5p targets BID. Data from TCGA show that miR-331-5p is significantly downregulated in TC compared to normal thyroids, while *BID* is overexpressed in TC tissues. High levels of *BID* occur in the thyroid tumors of TCGA in a Braf-like subtype, in metastatic tumors, and in the presence of nodal metastases.

In summary, as a result of this study, the miR-331-5p/BID axis was identified as a promising biomarker, offering new insights into TC progression mechanisms.

## Data Availability

The datasets generated in the label-free proteomic analysis are available via the ProteomeXchange with identifier PXD039884. Transcriptomics thyroid tissue data are available from The Cancer Genome Atlas (https://portal.gdc.cancer.gov/projects/TCGA-THCA) accessed on the 18 December 2023.

## Supplementary Materials

The following supporting information can be downloaded at: https://www.mdpi.com/article/10.3390/biomedicines12030658/s1, Figure S1: Ectopic expression of miR-331-5p in TC transfected cells. Figure S2: Silencing of miR-331-5p in TC transfected cells. Figure S3: Effects of the miR-331-5p modulation on the proliferation of TC stably transfected cells. Figure S4: Effects of the miR-331-5p modulation on the proliferation of TC transiently transfected cells. Figure S5: Effects of miR-331-5p silencing on the invasive phenotype of 8505C cell line. Figure S6: The miR-331-5p binding site on 3′UTR of BID mRNA predicted by online tools. Table S1: List of proteins differentially expressed in CAL62/miR-331-5p versus CAL62/miR-Null identified by label-free proteomic screening.
