# Supplementary material for: miR-331-5p Affects Motility of Thyroid Cancer Cell Lines and Regulates BID Expression
Source: Biomedicines. 2024 Mar 15;12(3):658. doi: 10.3390/biomedicines12030658 (PMC10968654; doi:10.3390/biomedicines12030658)
Supplement: Supplementary file 1 [file biomedicines-12-00658-s001.zip › biomedicines-2682292-supplementary.pdf]

Supplementary Materials

Supplementary Figures

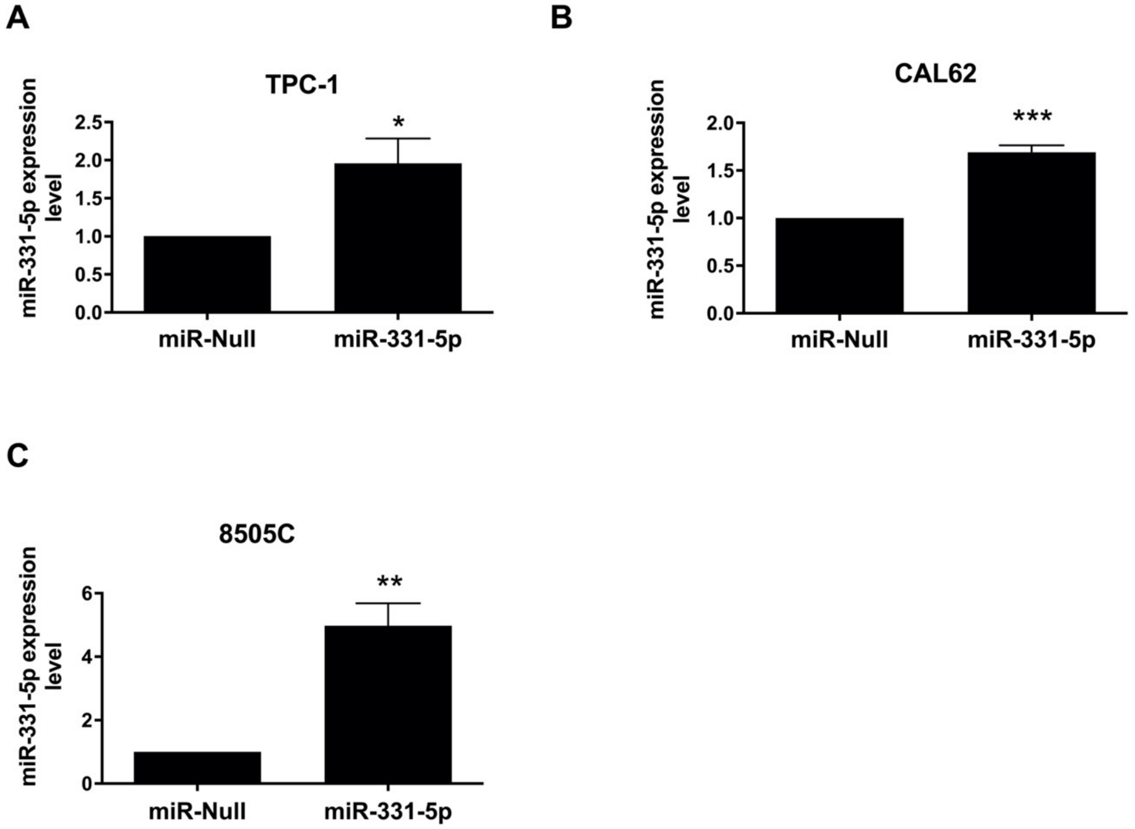

Figure S1. Ectopic expression of miR-331-5p in TC transfected cells. q-RT-PCR was performed to analyze the expression level of miR-331-5p in the mass population obtained from TPC-1 (**A**) and CAL62 (**B**) stably transfected with a plasmid expressing the precursor of miR-331-5p or with miRNA scrambled control (miR-Null). **C**. Expression level of miR-331-5p in 8505C cells after 48 hours of transient transfection with the above-mentioned plasmids. Each q-RT-PCR was performed in triplicates in three different RNA preparations obtained from the same mass population (**A-B**) or from three independent transient transfections (**C**). The data are presented as mean ± SEM. *, $p < 0.05$; **, $p < 0.01$; ***, $p < 0.001$.

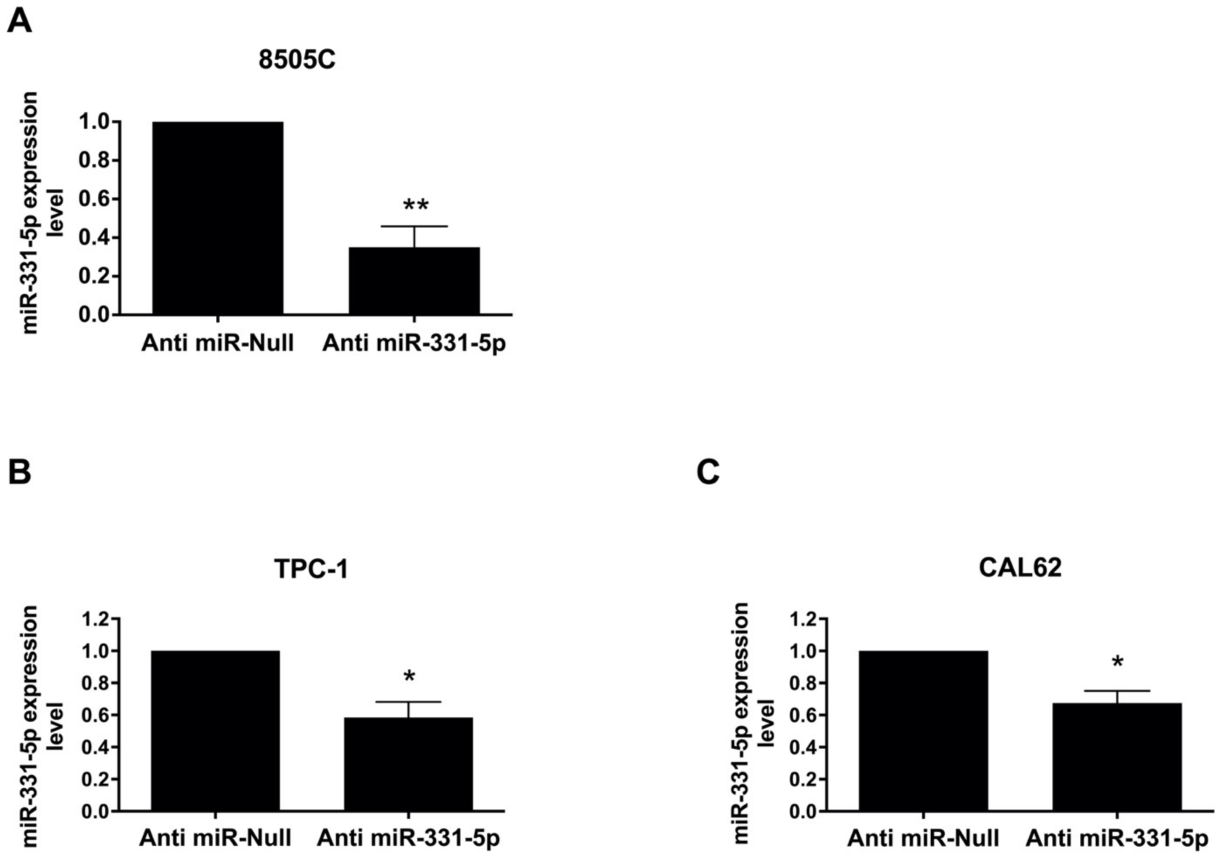

Figure S2. Silencing of miR-331-5p in TC transfected cells. Expression level of miR-331-5p was measured by q-RT-PCR in 8505C cells (**A**) stably transfected with plasmid inhibiting miR-331-5p (Anti miR-331-5p) and with miRNA inhibitor scrambled control plasmid (Anti miR-Null), in TPC-1 (**B**) and in CAL62 (**C**) cells transiently transfected with the above-mentioned plasmids for 48 hours. Each q-RT-PCR was performed in triplicates in three different RNA preparations obtained from the same mass population (**A**) or from RNA of three independent transient transfections (**B-C**). The data are presented as mean ± SEM. *, $p < 0.05$; **, $p < 0.01$.

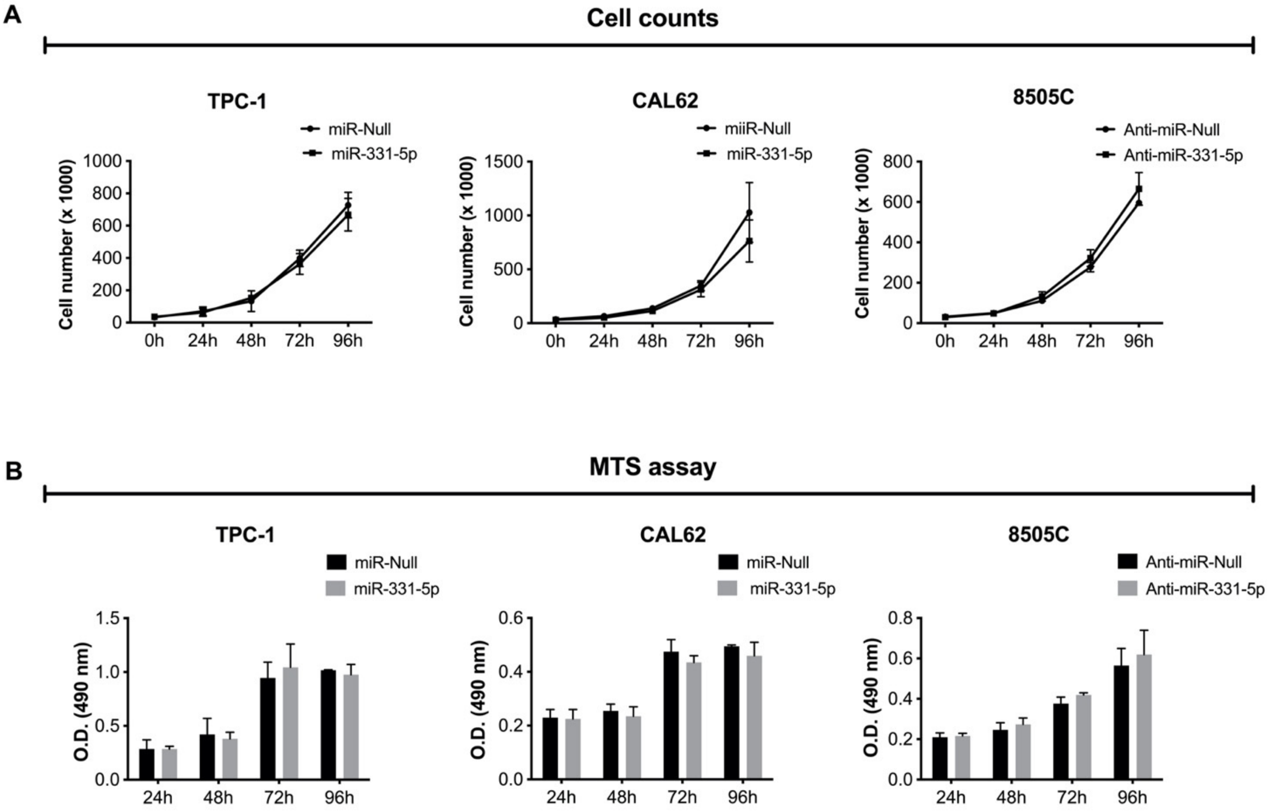

Figure S3. Effects of the miR-331-5p modulation on the proliferation of TC stably transfected cells. A. Stably transfected cells were plated (0 h) and counted at different time points. **B.** Cell proliferation was also assessed by MTS assay. MTS solution was added to each well, at the indicated time points, and the optical density (O.D.) was read at 490 nm. All experiments were performed three time in triplicates and results are reported as mean ± SEM.

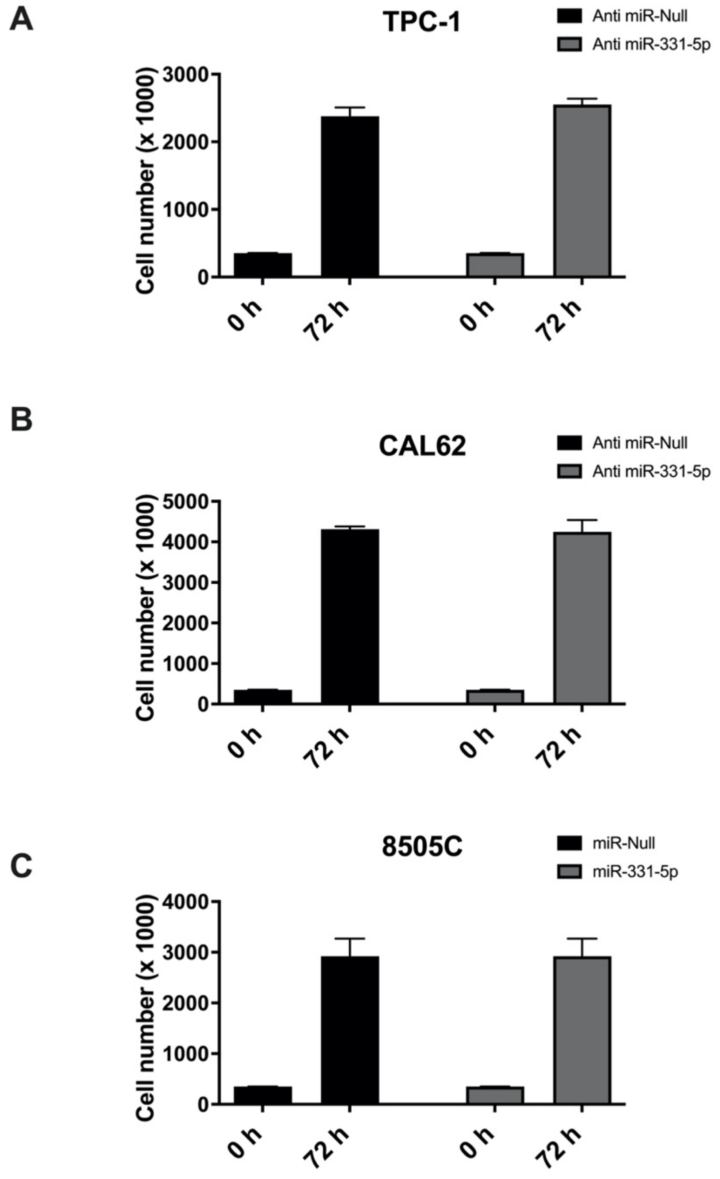

Figure S4. Effects of the miR-331-5p modulation on the proliferation of TC transiently transfected cells. At the end of wound closure assay, TPC-1 (**A**), CAL62 (**B**) and 8505C (**C**) cells transiently transfected with the indicated plasmids were collected by trypsinization and counted using TC10 automated cell counter. All experiments were performed three time and results are reported as mean ± SEM.

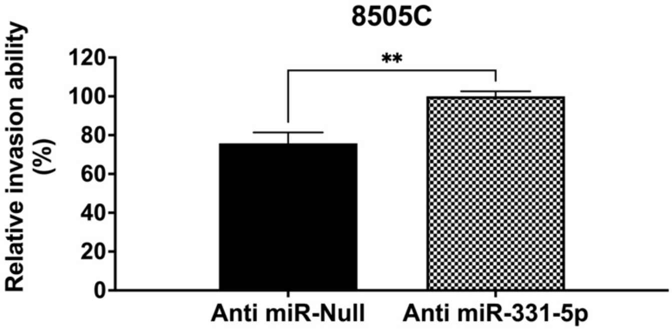

Figure S5. Silencing of miR-331-5p affects the invasive phenotype of 8505C cell line. The invasive capability of 8505C cells was analyzed *in vitro* by plating the cells on the Matrigel Matrix-coated permeable supports (8.0 µm pore diameter). After the incubation time, cells were colored in crystal violet and quantified by eluting the colorant and the spectrophotometric lecture (O.D. 550 nm). Data, shown as percentage, represent the mean ± SEM. **, $p < 0.01$.

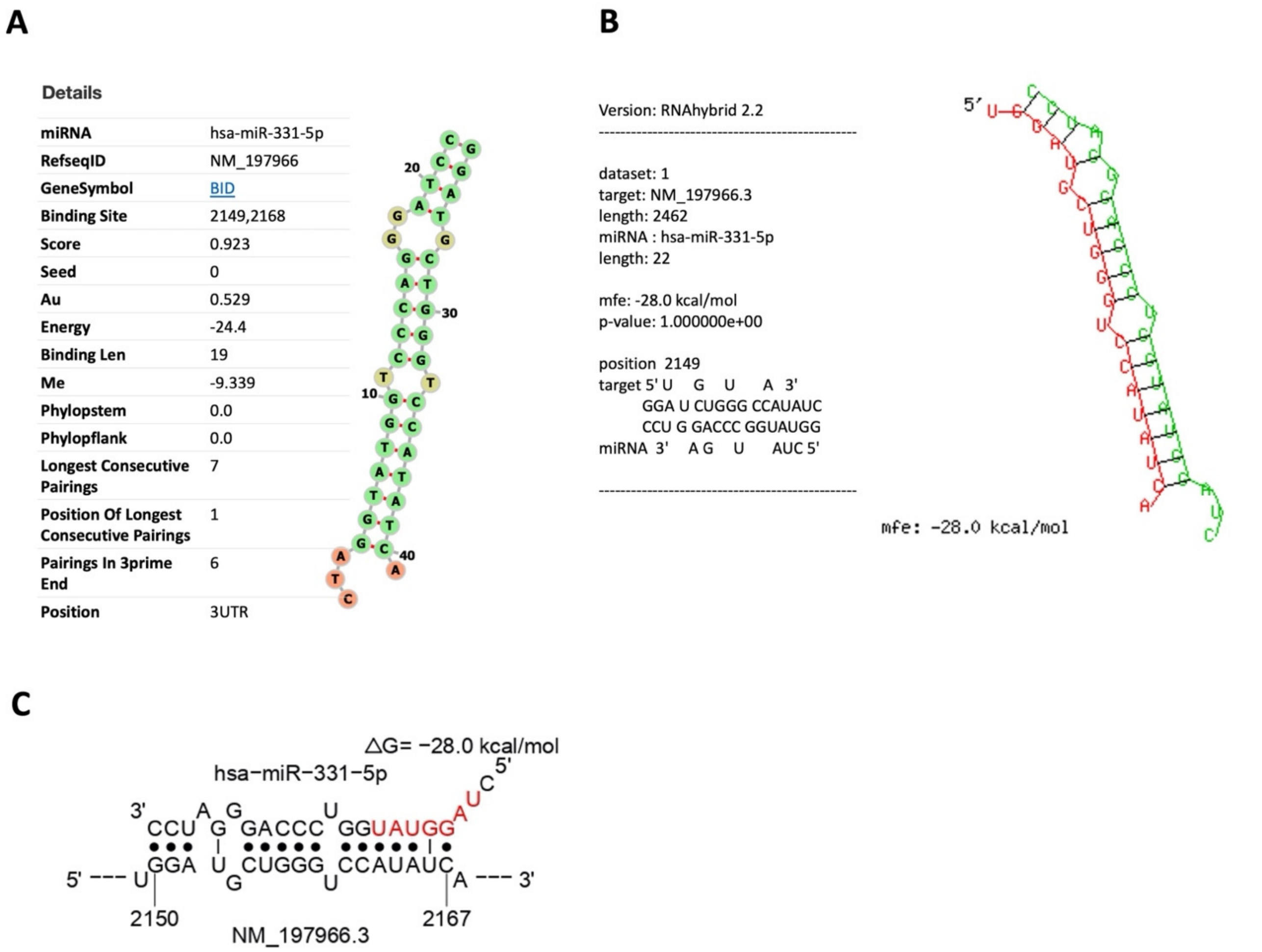

Figure S6. The miR-331-5p binding site on 3'UTR of BID mRNA predicted by online tools. The details and the secondary structure of the miR-331-5p and BID RNA-duplex predicted by miRWalk (**A**) on 27 December 2023 and by RNAhybrid (**B**) on 11 January 2024. **C**. Hybrid diagram for a binding non-canonical site between hsa-miR-331-5p and BID predicted by STarMir on 11 January 2024.

Supplementary Table

Table S1: List of proteins differentially expressed in CAL62/miR-331-5p *versus* CAL62/miR-Null identified by label-free proteomic screening.

Accession	Protein	*p*-value	Fold Change
P10620	Microsomal glutathione S-transferase 1 OS=Homo sapiens GN=MGST1 PE=1 SV=1 – [MGST1_HUMAN]	0.0281	6.83
Q9Y2T2	AP-3 complex subunit mu-1 OS=Homo sapiens GN=AP3M1 PE=1 SV=1 – [AP3M1_HUMAN]	0.0023	6.13
P42765	3-ketoacyl-CoA thiolase, mitochondrial OS=Homo sapiens GN=ACAA2 PE=1 SV=2 – [THIM_HUMAN]	0.0389	5.35
P53985	Monocarboxylate transporter 1 OS=Homo sapiens GN=SLC16A1 PE=1 SV=3 – [MOT1_HUMAN]	0.0298	4.85
Q9Y5L0	Transportin-3 OS=Homo sapiens GN=TNPO3 PE=1 SV=3 – [TNPO3_HUMAN]	0.0003	4.57
Q8N392	Rho GTPase-activating protein 18 OS=Homo sapiens GN=ARHGAP18 PE=1 SV=3 – [RHG18_HUMAN]	0.0071	3.44
P49841	Glycogen synthase kinase-3 beta OS=Homo sapiens GN=GSK3B PE=1 SV=2 – [GSK3B_HUMAN]	0.0072	3.10
Q8WXE9	Stonin-2 OS=Homo sapiens GN=STON2 PE=1 SV=1 – [STON2_HUMAN]	0.0051	2.89
P49427	Ubiquitin-conjugating enzyme E2 R1 OS=Homo sapiens GN=CDC34 PE=1 SV=2 – [UB2R1_HUMAN]	0.0119	2.69
Q96E29	Transcription termination factor 3, mitochondrial OS=Homo sapiens GN=MTERF3 PE=1 SV=2 – [MTEF3_HUMAN]	0.0197	2.60
Q6UW02	Cytochrome P450 20A1 OS=Homo sapiens GN=CYP20A1 PE=2 SV=1 – [CP20A_HUMAN]	0.0407	2.42
P82675	28S ribosomal protein S5, mitochondrial OS=Homo sapiens GN=MRPS5 PE=1 SV=2 – [RT05_HUMAN]	0.0163	2.39
P20292	Arachidonate 5-lipoxygenase-activating protein OS=Homo sapiens GN=ALOX5AP PE=1 SV=2 – [AL5AP_HUMAN]	0.0118	2.31
Q8NFH3	Nucleoporin Nup43 OS=Homo sapiens GN=NUP43 PE=1 SV=1 – [NUP43_HUMAN]	0.0046	2.29
Q96F85	CB1 cannabinoid receptor-interacting protein 1 OS=Homo sapiens GN=CNRIP1 PE=1 SV=1 – [CNRP1_HUMAN]	0.0132	2.10
Q9Y2Z4	Tyrosine--tRNA ligase, mitochondrial OS=Homo sapiens GN=YARS2 PE=1 SV=2 – [SYYM_HUMAN]	0.0025	2.05
Q9Y606	tRNA pseudouridine synthase A, mitochondrial OS=Homo sapiens GN=PUS1 PE=1 SV=3 – [TRUA_HUMAN]	0.0184	1.99
Q15643	Thyroid receptor-interacting protein 11 OS=Homo sapiens GN=TRIP11 PE=1 SV=3 – [TRIPB_HUMAN]	0.0249	1.92
Q6P9B6	TLD domain-containing protein 1 OS=Homo sapiens GN=TLDC1 PE=1 SV=2 – [TLDC1_HUMAN]	0.0014	1.88
Q96A49	Synapse-associated protein 1 OS=Homo sapiens GN=SYAP1 PE=1 SV=1 – [SYAP1_HUMAN]	0.0051	1.82

Q9NX20	39S ribosomal protein L16, mitochondrial OS=Homo sapiens GN=MRPL16 PE=1 SV=1 – [RM16_HUMAN]	0.0375	1.71
Q9NYK5	39S ribosomal protein L39, mitochondrial OS=Homo sapiens GN=MRPL39 PE=1 SV=3 – [RM39_HUMAN]	0.0057	1.64
P43121	Cell surface glycoprotein MUC18 OS=Homo sapiens GN=MCAM PE=1 SV=2 – [MUC18_HUMAN]	0.0024	1.60
P04183	Thymidine kinase, cytosolic OS=Homo sapiens GN=TK1 PE=1 SV=2 – [KITH_HUMAN]	0.0277	1.48
Q9P2T1	GMP reductase 2 OS=Homo sapiens GN=GMPR2 PE=1 SV=1 – [GMPR2_HUMAN]	0.0108	1.48
Q9Y248	DNA replication complex GINS protein PSF2 OS=Homo sapiens GN=GINS2 PE=1 SV=1 – [PSF2_HUMAN]	0.0367	1.48
Q86U90	Yrdc domain-containing protein, mitochondrial OS=Homo sapiens GN=YRDC PE=1 SV=1 – [YRDC_HUMAN]	0.0097	1.31
P46783	40S ribosomal protein S10 OS=Homo sapiens GN=RPS10 PE=1 SV=1 – [RS10_HUMAN]	0.0460	1.26
P35637	RNA-binding protein FUS OS=Homo sapiens GN=FUS PE=1 SV=1 – [FUS_HUMAN]	0.0258	1.25
Q9Y3B2	Exosome complex component CSL4 OS=Homo sapiens GN=EXOSC1 PE=1 SV=1 – [EXOS1_HUMAN]	0.0496	1.24
Q8WUP2	Filamin-binding LIM protein 1 OS=Homo sapiens GN=FBLIM1 PE=1 SV=2 – [FBLI1_HUMAN]	0.0029	1.22
Q9Y6G5	COMM domain-containing protein 10 OS=Homo sapiens GN=COMMD10 PE=1 SV=1 – [COMDA_HUMAN]	0.0229	-1.28
P55957	BH3-interacting domain death agonist OS=Homo sapiens GN=BID PE=1 SV=1 – [BID_HUMAN]	0.0018	-1.31
Q86VM9	Zinc finger CCCH domain-containing protein 18 OS=Homo sapiens GN=ZC3H18 PE=1 SV=2 – [ZCH18_HUMAN]	0.0139	-1.95
Q9BYT8	Neurolysin, mitochondrial OS=Homo sapiens GN=NLN PE=1 SV=1 – [NEUL_HUMAN]	0.0031	-2.06
Q9UPU5	Ubiquitin carboxyl-terminal hydrolase 24 OS=Homo sapiens GN=USP24 PE=1 SV=3 – [UBP24_HUMAN]	0.0371	-2.76
P98196	Probable phospholipid-transporting ATPase IH OS=Homo sapiens GN=ATP11A PE=1 SV=3 – [AT11A_HUMAN]	0.0473	-3.55
Q7Z3B4	Nucleoporin p54 OS=Homo sapiens GN=NUP54 PE=1 SV=2 – [NUP54_HUMAN]	0.0121	-3.79
Q9UQN3	Charged multivesicular body protein 2b OS=Homo sapiens GN=CHMP2B PE=1 SV=1 – [CHM2B_HUMAN]	0.0023	-6.87
